# Selinexor synergizes with dexamethasone to repress mTORC1 signaling and induce multiple myeloma cell death

**DOI:** 10.18632/oncotarget.25368

**Published:** 2018-05-22

**Authors:** Christian Argueta, Trinayan Kashyap, Boris Klebanov, Thaddeus J. Unger, Cathy Guo, Susie Harrington, Erkan Baloglu, Margaret Lee, William Senapedis, Sharon Shacham, Yosef Landesman

**Affiliations:** ^1^ Karyopharm Therapeutics Inc, Newton, MA 02459, USA

**Keywords:** exportin-1, selinexor, glucocorticoid receptor, mTOR

## Abstract

Multiple myeloma (MM) is a plasma cell neoplasm that results in over 11,000 deaths in the United States annually. The backbone therapy for the treatment of MM patients almost always includes combinations with corticosteroids such as dexamethasone (DEX). We found that DEX in combination with selinexor, an inhibitor of exportin-1 (XPO1) activity, synergistically inhibits the mTOR pathway and subsequently promotes cell death in MM cells. Specifically, we show that selinexor induces the expression of the glucocorticoid receptor (GR) and when combined with dexamethasone increases GR transcriptional activity. Moreover, we found that key downstream targets of the mTOR pathway are deregulated by the combination and identified a mechanism in which GR enhances the expression of REDD1 in GR positive cells while suppressing mTOR activity and cell viability. While the single agent activity of selinexor in MM cells appears to be GR-independent, synergy with DEX depends on GR expression. These data suggest that patients with tumor cells that are GR positive will benefit substantially from the combination. The current findings are consistent with the beneficial therapeutic outcome in patients with MM when treated with the combination of selinexor and DEX. In addition, they provide a rationale for testing GR and REDD1 as predictive and prognostic markers of response, respectively, for patients treated with this beneficial combination.

## INTRODUCTION

Multiple myeloma (MM) is an incurable neoplasm of plasma cell origin that is generally contained in the bone marrow and leads to hypercalcemia, renal failure, anemia, and bone lesions [1]. Despite significant improvements in the treatment and the overall survival of patients with MM, the median overall survival is only 4–7 years after initial diagnosis [2–4]. Molecular and epigenetic alterations such as chromosomal translocations, deregulated signal transduction pathways, and changes in the bone marrow microenvironment have been shown to contribute to myeloma progression and resistance to therapy [5]. Due to the absence of a central genetic event driving disease manifestation, current efforts have focused on identifying changes in gene expression and understanding key signaling events deregulated in myeloma plasma cells.

Exportin-1 (XPO1 or CRM1) is a member of the karyopherin family of nuclear transport proteins, which actively shuttle large (> 40 kDa) macromolecules between the nucleus and cytoplasm through the nuclear pore complex embedded in the nuclear membrane. XPO1 is the sole exporter of over 200 known cargos, including the majority of tumor suppressor proteins (TSPs), as well as mRNAs, rRNAs, and snRNAs [6]. In cancers, including MM, XPO1 is frequently overexpressed, leading to the enhanced nuclear export of TSPs [7–14]. Overexpression of XPO1 allows cancer cells to evade genome surveillance and cell cycle regulation because nuclear localization is critical for the function of many TSPs [15]. Furthermore, higher levels of XPO1 expression are correlated with reduced overall survival in many cancers, including MM [7, 9, 16]. Therefore, XPO1 inhibition is an attractive therapeutic approach for the treatment of MM [12, 16]. Selinexor (KPT-330) is a first-in-class, orally bioavailable, Selective Inhibitor of Nuclear Export (SINE) that specifically binds to the Cys-528 residue located within the cargo binding pocket of XPO1. Inhibition of XPO1 disrupts the nuclear export of cargo proteins, resulting in the nuclear retention and functional reactivation of TSPs. Subsequently, SINE compound treatment leads to selective induction of intrinsic apoptotic pathways in malignant cells, while sparing normal cells [17].

Current therapy options for patients with relapse/refractory multiple myeloma (RRMM) almost always include the use of corticosteroids such as DEX, methylprednisolone or prednisone [2]. These steroids activate the glucocorticoid receptor (GR) and ultimately lead to apoptosis of MM cells through unknown signaling pathways [18]. DEX, a synthetic glucocorticoid (GC) with potent anti-inflammatory activity, is frequently administered as a direct chemotherapeutic agent in combination with other agents such as proteasome inhibitors (bortezomib or carfilzomib) or immunomodulatory drugs (pomalidomide or lenalidomide) in MM [2, 19, 20]. DEX and other GCs bind to monomeric GR proteins in the cytoplasm, the GC-GR complex undergoes a conformational change, forms a hyper-phosphorylated dimer, then translocates into the nucleus [21]. Once inside the nucleus, the GR complex recognizes glucocorticoid steroid response elements and recruits transcriptional machinery to promote or inhibit gene expression (transactivation or trans-repression) [22]. Although GCs are a cornerstone of MM therapy, resistance as well as progressive side effects can develop following prolonged exposure to DEX or other GCs emphasizing the need for novel therapies and combinations [23].

Selinexor, in combination with DEX (SEL-DEX) and other regimens, is currently being evaluated in the following clinical trials: (STORM NCT02336815, STOMP NCT02343042, BOSTON NCT03110562). The current trials rely on observations made during earlier phase clinical trials that have shown that the combination of SEL-DEX improves tolerability and significantly increases the response rate when compared to selinexor as a single agent [20]. Based on these promising studies, we examined the effects of selinexor alone and in combination with DEX in different MM cell lines including the extensively characterized cell lines, GR^+^ MM.1S and GR^null^ MM.1R, which parallel the progression of MM and were generated from a single patient with MM [24]. Our data shows that selinexor synergizes with DEX to induce apoptotic cell death in GR^+^ cells. We further show that selinexor alone enhances the expression and translation of GR, making cells more sensitive to DEX. Consistently, GR transcriptional activation is synergistically enhanced by the SEL-DEX combination. Mechanistically, we show that mTOR activity, which plays a central role in MM disease progression and therapeutic resistance, is mitigated by the SEL-DEX synergy [25–29]. Finally, the combination of SEL-DEX showed marked synergy in a MM xenograft model. These results suggest that, along with the single agent anti-MM activity, selinexor augments the activity of GCs and improves the overall response of patients with MM to this potent combination.

## RESULTS

### Synergistic cytotoxic effects of the SEL-DEX combination in cells expressing GR

**Figure 1:**
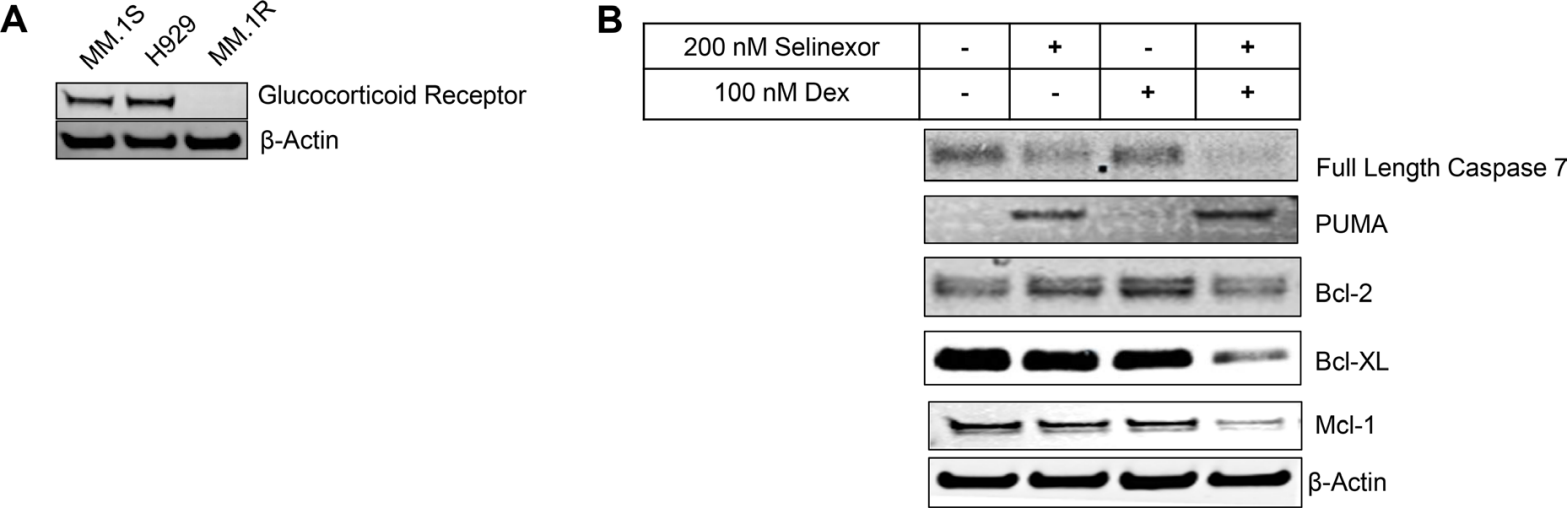
SEL-DEX synergizes to induce cell death in cells expressing GR (**A**) The expression of GR was evaluated by western blotting. (**B**) MM.1S cells were treated with 200 nM selinexor and 100 nM DEX for 24 hours and protein levels of Bcl-2 family members was analyzed by western blotting. The combination treatment led to reduction in the levels of the Bcl-2, Bcl-xL and Mcl-1 and up-regulation of PUMA. In addition, increased caspase-7 cleavage suggests that treatment with the combination kills cells via the intrinsic signaling pathway of programmed cell death.

DEX, and other GCs, are frequently given to cancer patients to treat inflammatory side effects incurred by other chemotherapy treatments and potentially enhance anti-tumor activity. In hematological malignancies, particularly MM, DEX is considered a cornerstone of therapy and can be administered as a single agent or in combination with other therapeutic agents [19]. Moreover, the STORM (NCT02336815) and STOMP (NCT02343042) clinical studies have demonstrated anti-myeloma activity when DEX is combined with selinexor in human patients [30]. Based on these findings, we examined whether DEX enhances the anticancer activity of selinexor in a GR dependent manner using GR^+^ (MM.1S and H929) and GR^null^ (MM.1R) MM cells lines (Figure 1A). We treated cells with selinexor or DEX, as single agents or in combination at various concentrations using serial titration and then examined cell viability after 72 hours of treatment. We found that sub-cytotoxic concentrations of DEX synergistically enhanced the cytotoxic activity of selinexor in GR^+^ MM cells (40 nM IC_50_ to 11 nM IC_50_ in MM.1S cells and 220 nM IC_50_ to 30 nM IC_50_ in H929 cells) (Table 1). Combination indices were determined using the Chou-Talaly method with the aid of CompuSyn software (Supplementary Tables 1 and 2) [31]. Consistently, the combination of sub-cytotoxic doses of DEX (100 nM at 24 hours) enhanced the expression of pro-apoptotic markers (PUMA and caspase 7 cleavage) and decreased pro-survival markers (Bcl-2, Bcl-xL and Mcl-1) when compared to selinexor alone (200 nM at 24 hours) in GR^+^ MM.1S (Figure 1B). As expected, DEX had no effect on the activity of selinexor or cell viability of GR^null^ MM.1R cells at all concentrations tested (up to 30 μM) (Table 1).

**Table 1:** Selinexor IC_50_

	Selinexor IC_50_	Selinexor IC_50_ (in the presence of Dexamethasone)	
		Dexamethasone @ IC_25_	10 μM Dexamethasone
**MM.1S**	40 nM	11 nM	< 0.15 nM
**H929**	220 nM	30 nM	4.6 nM
**MM.1R**	50 nM	N/A	57 nM

MM.1S, H929, MM.1R cells were treated with different concentrations of selinexor alone or in the presence of DEX at IC_25_ or 10 μM for 72 hrs. DEX at IC_25_ reduced the of IC_50_ selinexor’s by 3.7 fold and 7.3 fold for MM.1S and H929 cells, respectively, but had no effect on GR^null^ MM.1R cells even at 10 μM.

### Combination of SEL-DEX results in a synergistic increase in GR transcriptional activity

**Figure 2:**
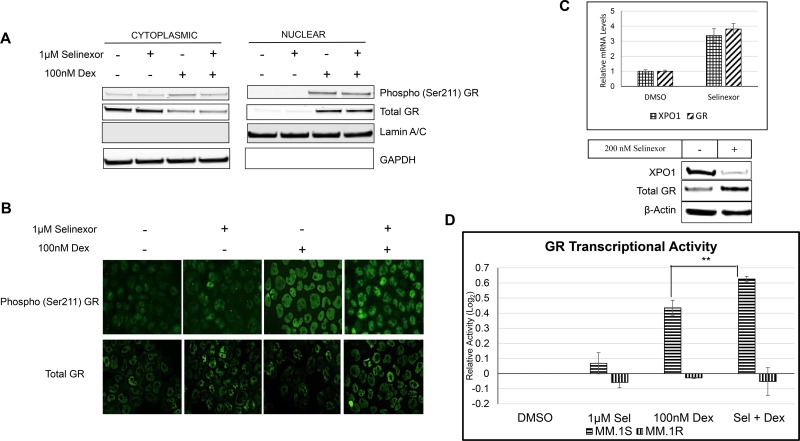
SEL-DEX results in a synergistic increase in GR transcriptional activity (**A**) Sub-cellular localization of Phospho (Ser-211) and total GR in MM.1S cells treated with 1 μM of selinexor and/or 100 nM DEX for 4 hours was evaluated by cellular fractionation and western blotting. The results that DEX, but not selinexor, induced phosphorylation of GR at Ser-211 resulting in GR nuclear localization. (**B**) Immunofluorescence staining of phospho and total GR in MM.1S cells treated with 1 μM of selinexor and/or 100 nM DEX for 4 hours. (**C**) MM.1S cells were treated with 200 nM of selinexor for 24 hours. The expression of XPO1 and GR was evaluated by real time PCR and western blotting. Selinexor increased GR mRNA and protein expression. (**D**) MM.1S and MM.1R cells were treated with 1 μM of selinexor and/or 100 nM DEX for 4 hours and GR transcriptional activity was measured by Affymetrix GR ELISA assay. In MM.1S cells, the combination resulted in a 4.3-fold increase in basal GR activity, compared to 2.7-fold increase by DEX treatment alone. GR^null^ MM.1R cells had no change in basal GR activity in the presence of the either compound alone or in combination.

Since selinexor inhibits XPO1-mediated nuclear export, we examined whether the synergistic effects of SEL-DEX on cell death were due to enhanced nuclear retention of GR. Although GR contains a putative nuclear export signal, previous experiments using the XPO1 inhibitor, leptomycin B, did not show nuclear retention of GR [32]. To determine whether selinexor treatment can enhance nuclear localization of GR, we performed nuclear fractionation and immunofluorescence experiments on GR^+^ MM.1S cells in the presence of selinexor or DEX alone or in combination. Our experiments confirm that selinexor does not affect the localization or activity of GR, as evidenced by phosphorylation of the Ser-211 residue of GR, which serves as a surrogate marker of structural rearrangement and ligand activation and is required for full GR transcriptional activity (Figure 2A–2B) [33]. It should be noted that only DEX treatment of cells promoted the nuclear translocation of GR.

However, we did find that selinexor treatment resulted in an increase in the total steady state protein levels of the GR in GR^+^ MM.1S (Figure 2B). The induction of GR expression is less evident in the heavily processed nuclear fractionation (Figure 1A). To address this discrepancy we examined GR expression in a quantitative fashion by analyzing total mRNA and protein expression in MM.1S cells treated with 200 nM selinexor (Figure 2C). Our results show that GR expression is induced by selinexor in MM.1S cells. This finding was also confirmed by examining GR mRNA expression across a panel of MM cell lines treated with selinexor (Supplementary Figure 1).

As previously reported, XPO1 inhibition by selinexor results in a decrease in XPO1 protein expression and a related increase in XPO1 mRNA by virtue of a positive feedback loop [34]. Having established an increase in total GR in the presence of selinexor, we sought to determine if GR transcriptional activity was altered in the presence of selinexor and DEX. Using a GR transcriptional activity ELISA assay that measures the binding of phosphorylated GR to a double stranded DNA sequence containing a GR consensus binding site, we found that selinexor treatment alone did not alter GR transcriptional activity while DEX treatment alone promoted an increase in transcriptional activity. Strikingly, the combination of SEL-DEX prompted an increase over basal activity and a 1.5-fold increase over DEX induced transcriptional activity (Figure 2D). Additionally, GR^null^ MM.1R cells had no change in basal GR activity in the presence of DEX or the SEL-DEX combination.

### Reverse phase protein array (RPPA) shows mTOR pathway genes dysregulated by the SEL-DEX combination

Having established a mechanistic link behind the synergistic effect observed when selinexor is used in combination with DEX, we next focused on finding which genes and pathways were activated or disrupted by the combination. Because SINE compounds also inhibit the nuclear export of certain mRNAs and therefore affect their translation, we focused on changes in protein levels and modifications in the combination treated sample using reverse phase protein array technology (RPPA) at the MD Anderson Core Facility. Proteins and post-translational modifications that differed from either vehicle or single agent samples were analyzed by the gene network and function predicting software GeneMANIA [35]. This method identified major changes in several pathways (e.g. insulin receptor signaling pathway and ErbB signaling pathway) following the combination treatment with a significant emphasis on the mechanistic target of rapamycin (*p*-value = 2.4 × 10^−16^), formerly mammalian target of rapamycin (mTOR), pathway (Supplementary Figure 2A). Further analysis revealed that the combination treatment reduced the phosphorylation of mTOR at Ser-2448 which is indicative of mTOR activity (Supplementary Figure 2B) [36]. Phosphorylation of 4E-BP1 (Ser-65) and P70-S6K (T389), which are well established hallmarks of mTOR catalytic activity, were also reduced [37]. Moreover, the phosphorylation of the p70S6K substrate RPS6 (Ser-235/236), which is a downstream component of the mTOR pathway and involved in protein synthesis, was also disrupted by the combination [38]. Changes in the expression and post-translational modification of mTOR pathway components were also observed (e.g. Rictor, TSC1, TSC2, and AMPK [Thr-172]).

### The SEL-DEX combination inhibits mTOR signaling through both GR-dependent and independent pathways

**Figure 3:**
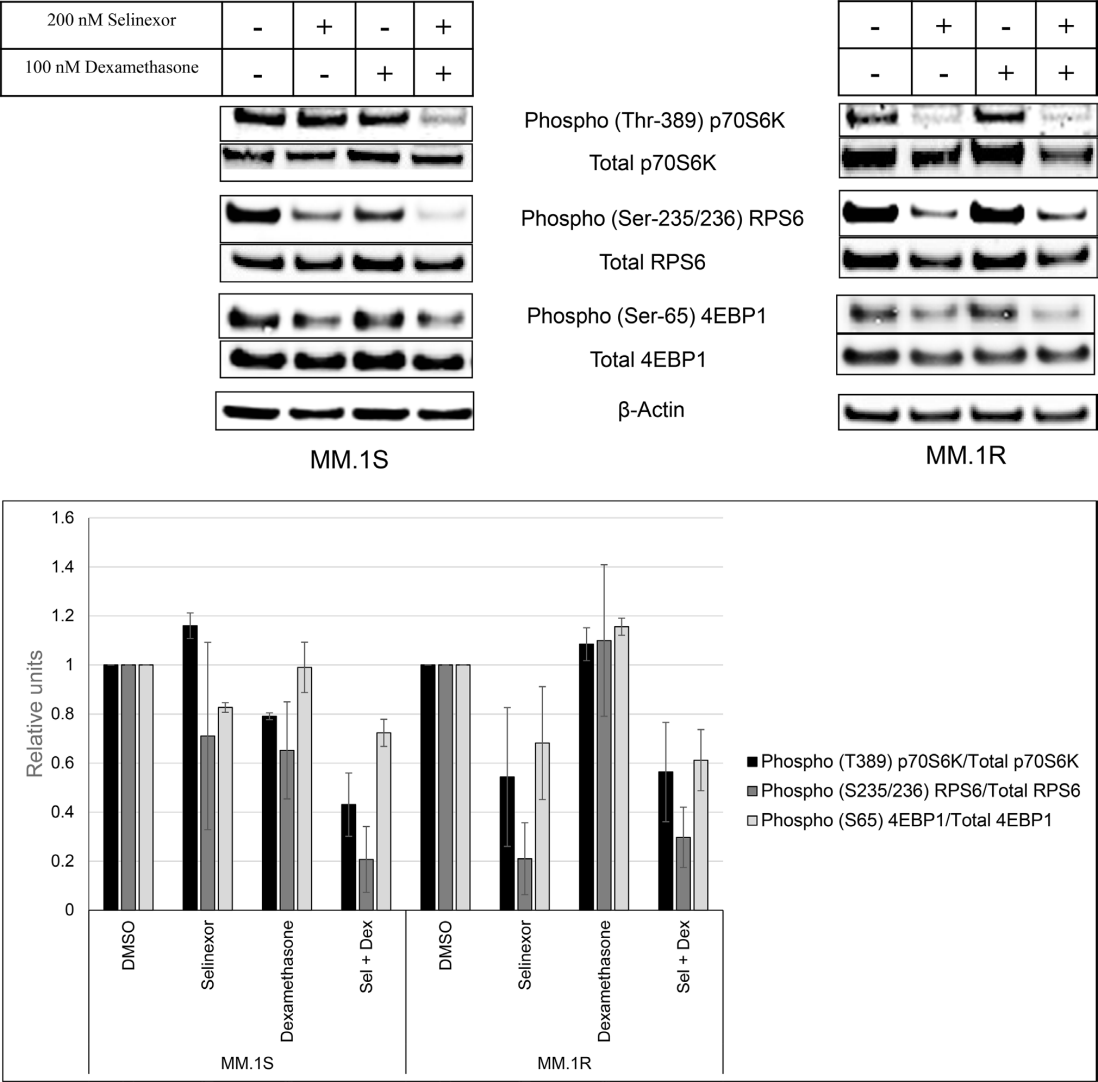
SEL-DEX inhibits the mTOR pathway MM.1S (GR^wt^) and MM.1R (GR^null^) cells were treated with 200 nM selinexor and 100 nM DEX for 24 hours. In MM.1S cells, selinexor alone had little effect on the mTOR pathway (p70S6K and p4EBP1). However, in combination with DEX, a significant reduction in p70S6K and 4EBP1 phosphorylation was seen. The phosphorylation levels of RPS6, downstream target of p70S6K, was also lowered more with the combination compared to the single agents. In MM.1R cells, the effect on the mTOR pathway was driven by selinexor with no effect from DEX.

To confirm the RPPA data suggesting a major role for the mTOR pathway in the observed synergy between SEL-DEX, we treated MM.1S cells with 100 nM DEX, 200 nM selinexor, or a combination of both compounds for 24 hours. The cells were then harvested and examined by western blot analysis (Figure 3). mTOR activity was assessed by quantifying the phosphorylation of p70S6K and 4E-BP1. Treatment with selinexor alone had little effect on 4E-BP1 and p70S6K phosphorylation. DEX at 100 nM was also ineffective at reducing mTOR activity. In contrast, the SEL-DEX combination significantly reduces phosphorylation of both p70S6K and 4E-BP1. Moreover, phosphorylation of the p70S6K substrate RPS6, was also reduced by the combination. It should be noted that single agent treatment with selinexor or DEX inhibited phosphorylation of RPS6.

As a control, the same modifications in the mTOR pathway were examined in GR^null^ MM.1R cells. Selinexor alone significantly reduced the phosphorylation of 4E-BP1 and p70S6K. No differences were observed in the DEX and the combination treated samples when compared to the vehicle and single agent selinexor samples respectively. These results support the RPPA data (Supplementary Figure 2) and suggest that mTOR activity in MM.1R cells is repressed by selinexor in the absence of GR activity.

### The SEL-DEX combination modulates the expression of GR regulated genes

Because selinexor increases basal expression of GR and the combination with DEX affects the mTOR pathway, we hypothesize that elevated GR activity is responsible for the synergistic effect on the mTOR activity. In order to understand how the SEL-DEX combination inhibits the mTOR pathway, we focused on the expression of known GR-regulated modulators of mTOR activity. These genes include DEPTOR and MNK2 which directly inhibit mTORC1 complex and eIF4E, respectively [39, 40]. In addition, SESN1, SESN2, and REDD1 inhibit mTORC1 by targeting the upstream TSC1/2-RHEB axis [41, 42]. Finally, KLF15, which indirectly regulates the levels of branch chain amino acids and thus mTOR activity through the expression of BCAT2 [43].

**Figure 4:**
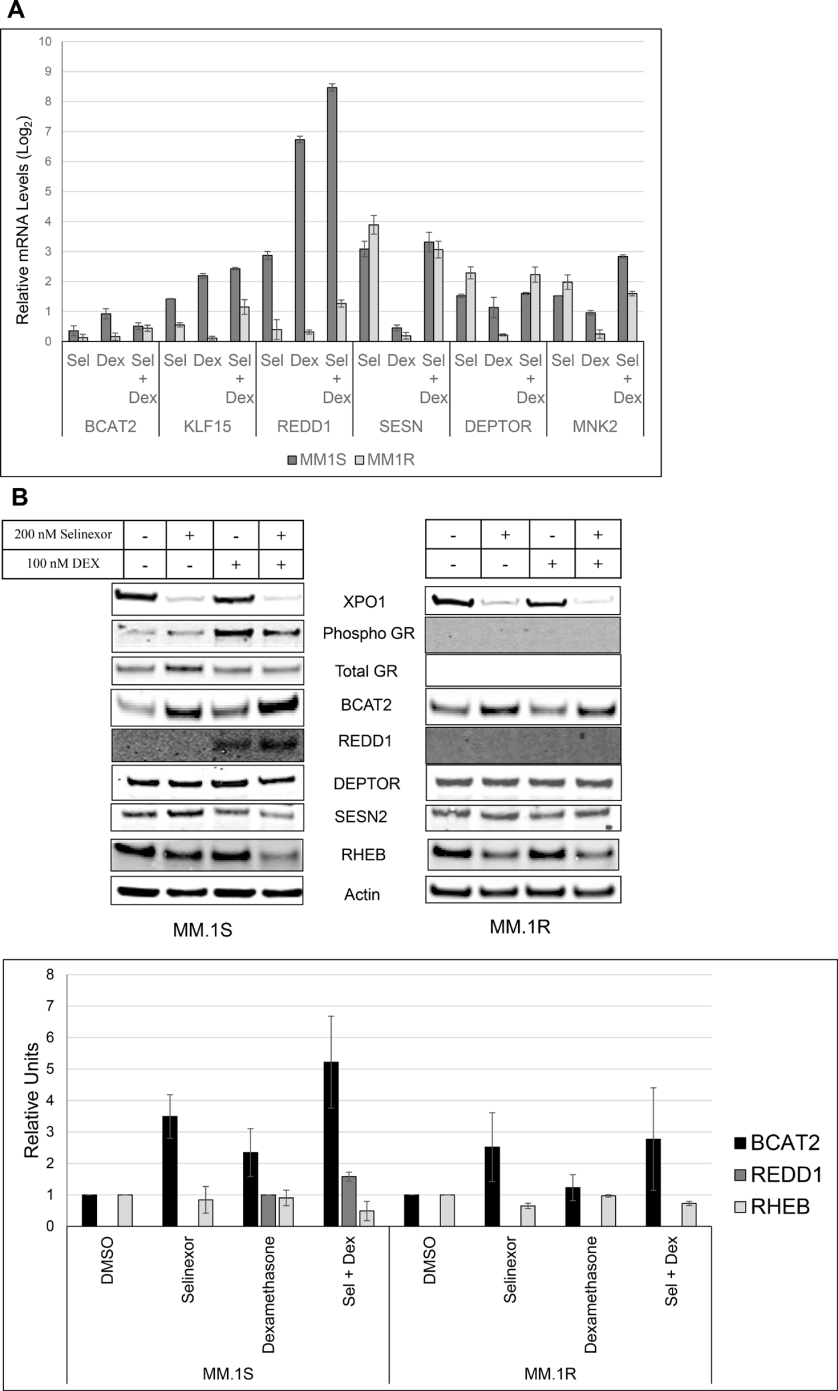
SEL-DEX modulates the expression of GR regulated genes MM.1S (GR^wt^) and MM.1R (GR^null^) cells were treated with 200 nM selinexor and 100 nM DEX for 24 hours. (**A**) The mRNA expression of the related genes was evaluated by real time PCR. DEX treatment alone induced the expression of all target genes to varying degrees in MM.1S cells, while having no effect on MM.1R cells. The combination with selinexor synergistically enhanced the expression of REDD1 in MM.1S cells. (**B**) The protein levels of GR regulated targets of mTOR activity was evaluated by western blotting. In MM.1S, BCAT2 and REDD1, both downstream of KLF15 (a GR target) exhibited combinatorial increases. In MM.1R, BCAT2 was significantly induced by selinexor treatment, although induction was not enhanced after adding DEX. RHEB expression was significantly reduced by the combination and selinexor in MM.1S and MM.1R respectively.

To determine the role these GR-regulated genes have on the mTOR pathway, we treated MM.1S and MM.1R cells with 100 nM of DEX, 200 nM of selinexor or a combination of both compounds for 24 hours. We then harvested the cells and examined gene expression. As expected, DEX induced the expression of all 6 genes in MM.1S cells while having no effect on MM.1R cells (Figure 4A). REDD1 in particular appears to be completely dependent on GR activity as the expression increased 100-fold in MM.1S cells while undetectable in MM.1R cells. Selinexor alone marginally induced the expression of all genes examined, except for KLF15 in MM.1R cells. The combination of SEL-DEX synergized dramatically to enhance the expression of REDD1 (4-fold compared to DEX treatment) in MM.1S cells, suggesting a prominent role in the regulation of mTOR activity.

### The SEL-DEX combination upregulates mTOR effector proteins REDD1 and BCAT2 in GR^+^ Cells

We evaluated the protein level of the GR-regulated (MNK2, DEPTOR, SESN, REDD1, KL15 and BCAT2) mTOR pathway modulators where mRNA expression changes were observed (Figure 4B). As described above, we treated MM.1S and MM.1R cells with 100 nM of DEX, 200 nM of selinexor, or a combination of both compounds for 24 hours. We then harvested the cells and examined protein expression by western blot analysis. Of the proteins examined, only BCAT2 and REDD1 expression increased in MM.1S cells treated with the combination when compared to treatment with either single agent. REDD1 expression was undetectable in vehicle and selinexor treated samples. Consistent with the qPCR data, DEX treatment induced the expression of REDD1, while the combination enhanced this expression. As expected, neither DEX treatment alone nor in combination with selinexor had any effect on REDD1 protein expression in MM.1R cells. On the other hand, selinexor did induce the expression of BCAT2. However, we discovered that BCAT2 functional activity was saturated by selinexor treatment alone and that MM.1R cells have significantly higher levels of BCAA when compared to MM.1S cells (data not shown). It should be noted that treatment with selinexor alone or in combination with dexamethasone resulted in a concomitant loss of RHEB expression in MM.1R and MM.1S cells, respectively.

### Silencing REDD1 reduces SEL-DEX inhibitory effect on mTOR activity

**Figure 5:**
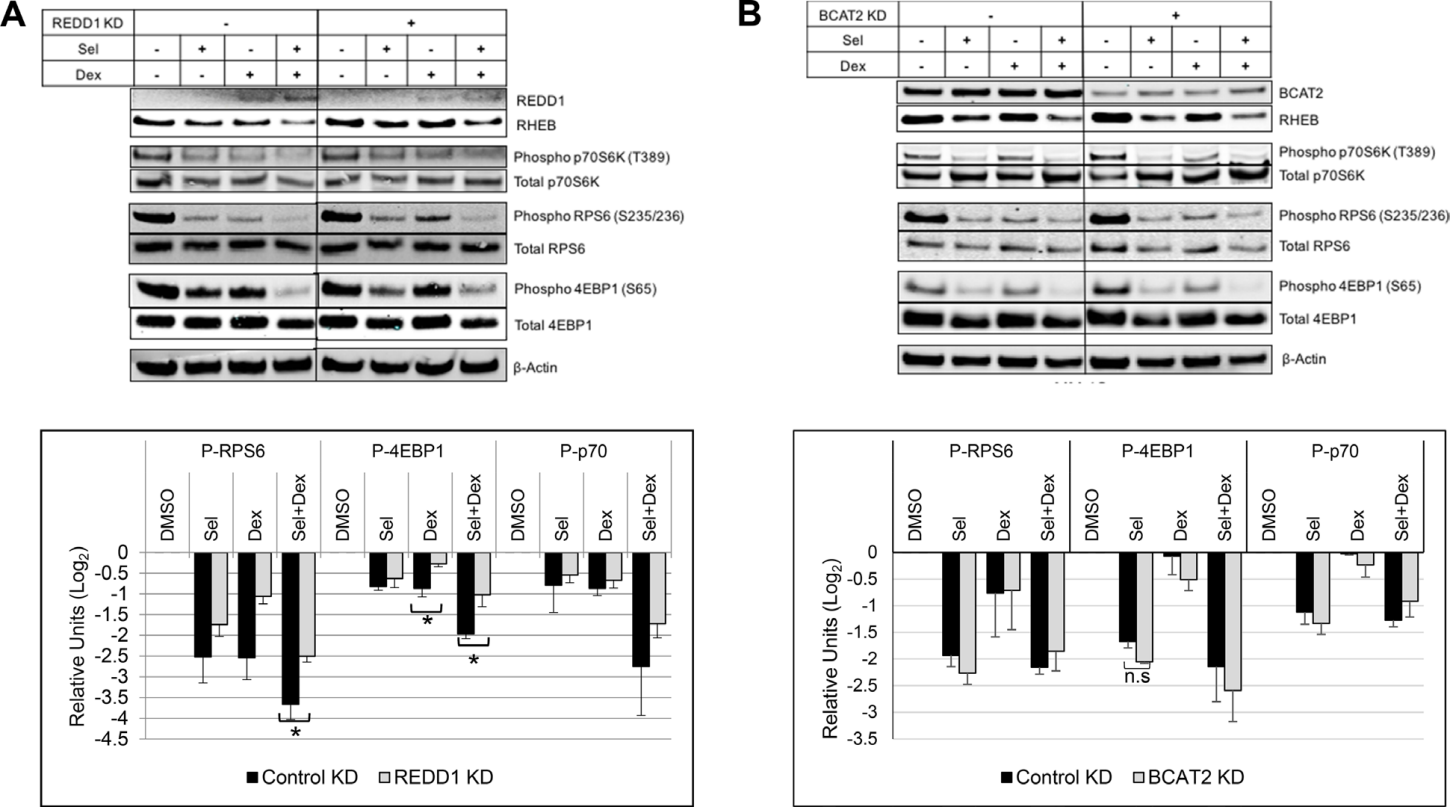
Silencing REDD1 reduces SEL-DEX inhibitory effect on mTOR-activity (**A**) MM.1S cells were transfected with either 40 nM REDD1 or control siRNA using Neon Transfection System as per manufacturer’s instruction. The siRNA transfected and non-transfected cells were treated for 24 hours with 200 nM selinexor and 100 nM DEX 48 hours post transfection. The cell lysates for evaluated by western blotting for the expression of mTOR pathway related genes. Densitometry analysis showed of statistically significant difference in the reduction of phospho (Ser-235/236) RPS6 and phospho (Ser-65) with the treatments between REDD1 silenced cells and cells transfected with control siRNA. (**B**) MM.1S cells were transfected with either 40 nM BCAT2 or control siRNA using Neon Transfection System and the cells were treated for 24 hours with 200 nM selinexor and 100 nM DEX for 48 hours post transfection. BCAT2 silencing didn’t significantly affect the impact of selinexor and DEX on mTOR targets.

Having established a synergistic induction of REDD1 and BCAT2 expression along with a concomitant loss of RHEB protein expression, we wanted to determine whether reducing REDD1 and BCAT2 could blunt the activity of the combination treatment. In order to evaluate this activity, we electroporated cells with two different REDD1 and BCAT2 siRNAs and selected the most efficient siRNAs for subsequent experiments (data not shown). The REDD1 or BCAT2 RNAi-exposed cells were then treated with selinexor or DEX alone or in combination (Figure 5). Mock transfected cells were used as a control. Despite our efforts we were unable to eliminate SEL-DEX related enhancement of BCAT2 expression. Similarly, although REDD1 expression is not detectable in the absence of DEX, REDD1 targeted RNAi did not completely eliminate the DEX or SEL-DEX related induction of REDD1 expression.

We showed that a reduction of REDD1 dampens the DEX or combination related induction of REDD1 resulting in mitigated reduction of RHEB expression and mTOR activity. Silencing REDD1 results in less 4E-BP1 inhibition (i.e. more phosphorylation) when compared to mock RNAi treated cells (Figure 5A). Although p-p70S6K levels do not appear to be affected, the phosphorylation of RPS6 is significantly reduced after REDD1 knockdown plus the combination treatment, suggesting that the marginal reduction of p-p70S6K is enough to reduce its effect on RPS6. In contrast, silencing BCAT2 does not appear to change the effect SEL-DEX treatment or selinexor alone has on the mTOR pathway in MM.1S (Figure 5B) or MM.1R (Supplementary Figure 3) cells, respectively.

### The SEL-DEX combination synergistically inhibits MM tumor growth *in vivo*

**Figure 6:**
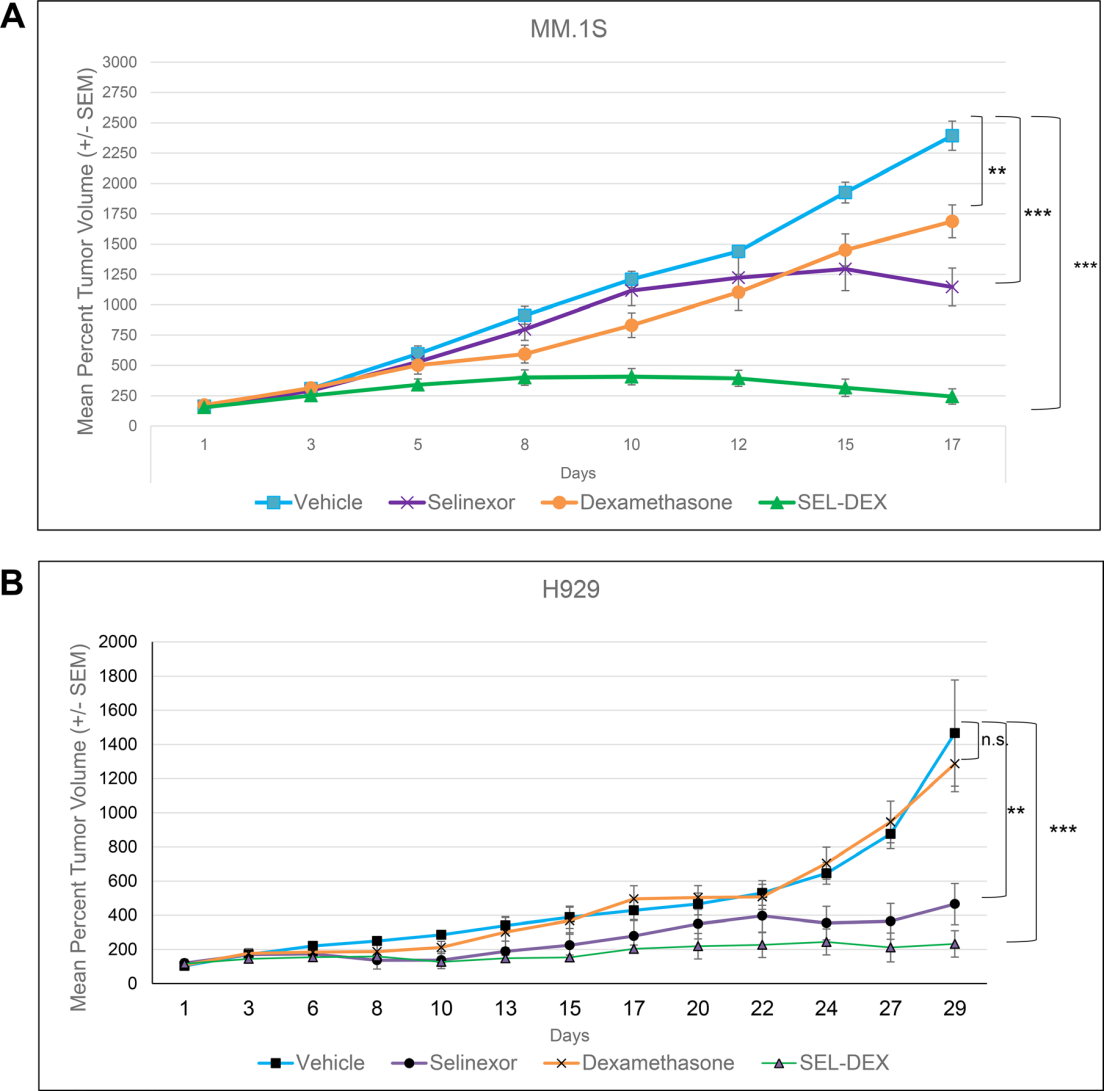
SEL-DEX synergistically inhibits MM tumor growth *in vivo* NOD-SCID mice were inoculated with MM.1S (**A**) or H929 (**B**) cells and allocated to one of four groups of eight or nine mice, respectively, such that mean tumor volume in each group was within the range of 108 to 113 mm^**3**^. Mice were treated with vehicle, 1 mg/kg DEX, 5 mg/kg of selinexor or the combination. Selinexor was given via oral gavage on a Monday-Wednesday-Friday schedule (MWF), while DEX was given via IP injection daily. The MM.1S group treated with DEX as single-agent therapy showed a significant reduction in tumor growth, while the H929 group that received DEX, showed no significant reduction in tumor growth compared to the vehicle treated group. The selinexor (*p* = 0.05) and SEL-DEX (*p* = 0.007) treated groups exhibited a significant reduction in tumor growth compared to the vehicle group in the MM.1S and H929 models.

To determine if the enhanced anti-cancer effects of the combination (see Figure 1 and Table 1) could be extended *in vivo*, we used a subcutaneous xenograft model of SCID mice harboring MM.1S or H929 GR^+^ myeloma tumors (Figure 6). Tumor bearing mice were treated with vehicle, selinexor (3 times/week), DEX (daily) or the combination of selinexor and DEX. The study was terminated once the average tumors in the vehicle treated group exceeded 1400 mm^3^. Single agent treatment of selinexor inhibited tumor growth when compared to the vehicle group in the MM.1S model (TGI 55%). MM.1S tumors extracted from mice treated with selinexor alone or vehicle were examined for GR expression by IHC. Our results confirm an induction of GR expression by selinexor in an *in vivo* setting (Supplementary Figure 4). Single agent treatment of DEX inhibited tumor growth by 32%, while the SEL-DEX group exhibited synergistic inhibition of tumor growth (TGI 96%) in the MM.1S model (Figure 6A). DEX had no effect on tumor size and proliferation in the H929 model (Figure 6B), which is consistent with our *in vitro* data (see Table 1), Selinexor treatment reduced tumor growth by 72%, while SEL-DEX combination treatment resulted in a 94% TGI compared to the vehicle group and a 65% TGI when compared to the selinexor group in the H929 model.

## DISCUSSION

This report describes the combinatorial effects between SEL-DEX in MM cells and elucidates a mechanism of action involving the suppression of mTOR activity. We found that the combination of SEL-DEX synergizes to induce apoptotic cell death in a GR dependent manner [24]. These results confirm our *in vitro* and clinical findings that DEX and selinexor synergistically inhibit cancer cell growth and raise the importance of dosing selinexor in the presence of DEX for the treatment of MM. Although GCs can have unwanted side effects (i.e. thinning skin, GI issues, mood changes, etc.), the absence of synergy in DEX resistant GR^null^ MM.1R cells suggests that the observed synergy is on target and dependent on GR status. The ability of SINE compounds to enrich nuclear localization of XPO1 cargo proteins prompted us to check the nuclear localization of GR in the presence of selinexor. The subcellular localization of GR, which contains both nuclear localization and export signals, has been shown to be dependent on nuclear import and export machinery, respectively [44, 45]. Although a role for XPO1 in the nucleocytoplasmic shuttling of GR has not been shown it cannot be ruled out [32]. We show that XPO1 inhibition by selinexor has no effect on the localization or phosphorylation/activation of GR. As expected, the GR ligand, DEX, induced the activation and nuclear translocation of GR in DEX and SEL-DEX treated cells.

Although GR activation, localization, and transcriptional activity of GR was not affected by selinexor alone, the transcriptional activity of GR was synergistically enhanced in the presence of DEX. Strikingly, we show, for the first time, that selinexor enhances the transcription and translation of GR. Together these data suggest that selinexor, which does not increase basal GR activity, increases total GR levels, while DEX activates GR leading to a synergistic enhancement of GRs anti-tumor transcriptional activity and cell death. The selinexor-induced enhancement of GR expression can be explained by SINE compound promoted nuclear accumulation of various TSPs and regulators of transcription, including regulators of GR (e.g. p53) [46]. It is well known that the level of GR expression is an important determining factor of the magnitude of cellular response to GC [47]. In fact, several clinical studies have shown that decreased GC sensitivity is associated with lower GR expression [48–52]. Our results suggest that patients still responding to steroid therapy would benefit from selinexor treatment, while patients with reduced GR expression could be re-sensitized to DEX in the presence of selinexor [53]. Moreover, our results indicate that patients lacking GR activity may be particularly sensitive to selinexor therapy.

It is well known that myeloma cell survival becomes dependent on key factors that contribute to their growth and proliferation. For example, cytokines such as interleukin 6 (IL-6), activate the JAK/STAT pathways and mitogen activated protein kinases (MAPKs) and ultimately lead to increased cell proliferation [54]. There is also evidence emphasizing the importance of the NF-κB pathway in MM progression along with reports highlighting the inhibitory properties of DEX on NF-κB activity [55–57]. We have previously shown that NF-κB activity is also negatively regulated by the combination of selinexor and proteasome inhibitors in MM cells [58, 59]. These findings contributed to the rationale supporting a pivotal randomized Phase 3 study (NCT03110562), which is evaluating selinexor in combination with bortezomib and low-dose DEX in patients with MM who have had one to three prior lines of therapy. Another such pathway is the PI3K/AKT pathway. Although mutations in PI3K/AKT genes are not common, this pathway is frequently activated in the majority of patients with MM [60]. In addition, mutations and dysregulation of RAS, PTEN, FGF, c-Myc, and CDKN signaling genes are often associated with MM progression [39, 61–67]. These pathways converge with the mTOR pathway, which is constituently active in myeloma, suggesting a functionally important role for mTOR in myeloma progression. mTOR, a member of the PI3K family of kinases, is the catalytic subunit of two structurally diverse atypical serine threonine kinase complexes (mTORC1 and mTORC2) located up and downstream of AKT and upstream of p70S6K, with functional processes ranging from autophagy, cell motility, cell proliferation, protein synthesis and transcription [68]. Our studies indicate that the combination of SEL-DEX predominately disrupts mTORC1 (i.e. mTOR, Raptor, GβL and DEPTOR); a nutrient sensor and a master regulator of cellular growth and proliferation [69].

Previous studies have shown that GR activity can inhibit mTORC1 through several potential GR targets. These include Sestrin, Deptor, REDD1, KLF15, BCAT2, and MKNK2 [39–43]. Notably many of these genes affect the tuberous sclerosis protein complex (TSC1/TSC2)-Ras homolog enriched in brain (RHEB) axis downstream of PI3K/AKT [70, 71]. Because selinexor induces the expression and transcriptional activity of GR, we postulated that elevated and activated GR protein could be inhibiting mTORC1 activity though one of the previously reported GR regulated mechanisms and that these increases in GR transcriptional activity would be detectable downstream by examining RHEB. Here, we report a loss of RHEB protein expression prompted by the SEL-DEX combination in GR^+^ cells. Our results show that the SEL-DEX combination synergizes to induce the transcription of several of these genes in GR dependent manner, however, only BCAT2 and REDD1 translation is positively affected. We also show that selinexor can increase BCAT2 protein levels in a GR independent manner, which could explain why selinexor inhibits the mTOR pathway in the absence of GR activity or REDD1 expression. Furthermore, silencing REDD1 expression, but not BCAT2, in GR^+^ cells significantly reduced the SEL-DEX induced loss of mTOR kinase activity in GR^+^ MM cells. However, it is worth noting that our inability to completely silence BCAT2 expression in these cells may account for the inability to preserve mTOR kinase activity following SEL-DEX treatment. In contrast, GR^null^ cells appear to lack REDD1 altogether and only BCAT2 expression is induced in a selinexor dependent manner. Together, these data suggest that BCAT2 might play a prominent role in the regulation of mTOR activity in the absence of GR transcriptional activity, while the GC related induction of REDD1 combines with BCAT2 activity to reduce mTOR activity in cells expressing GR. In addition, our results further confirm the reciprocal relationship between GR activity and BCAA levels, as GR^null^ MM.1R cells have higher levels of BCAAs when compared to GR^+^ MM.1S cells, which could suggest that cells can become more dependent on BCAA levels in the absence of GR activity [43].

**Figure 7:**
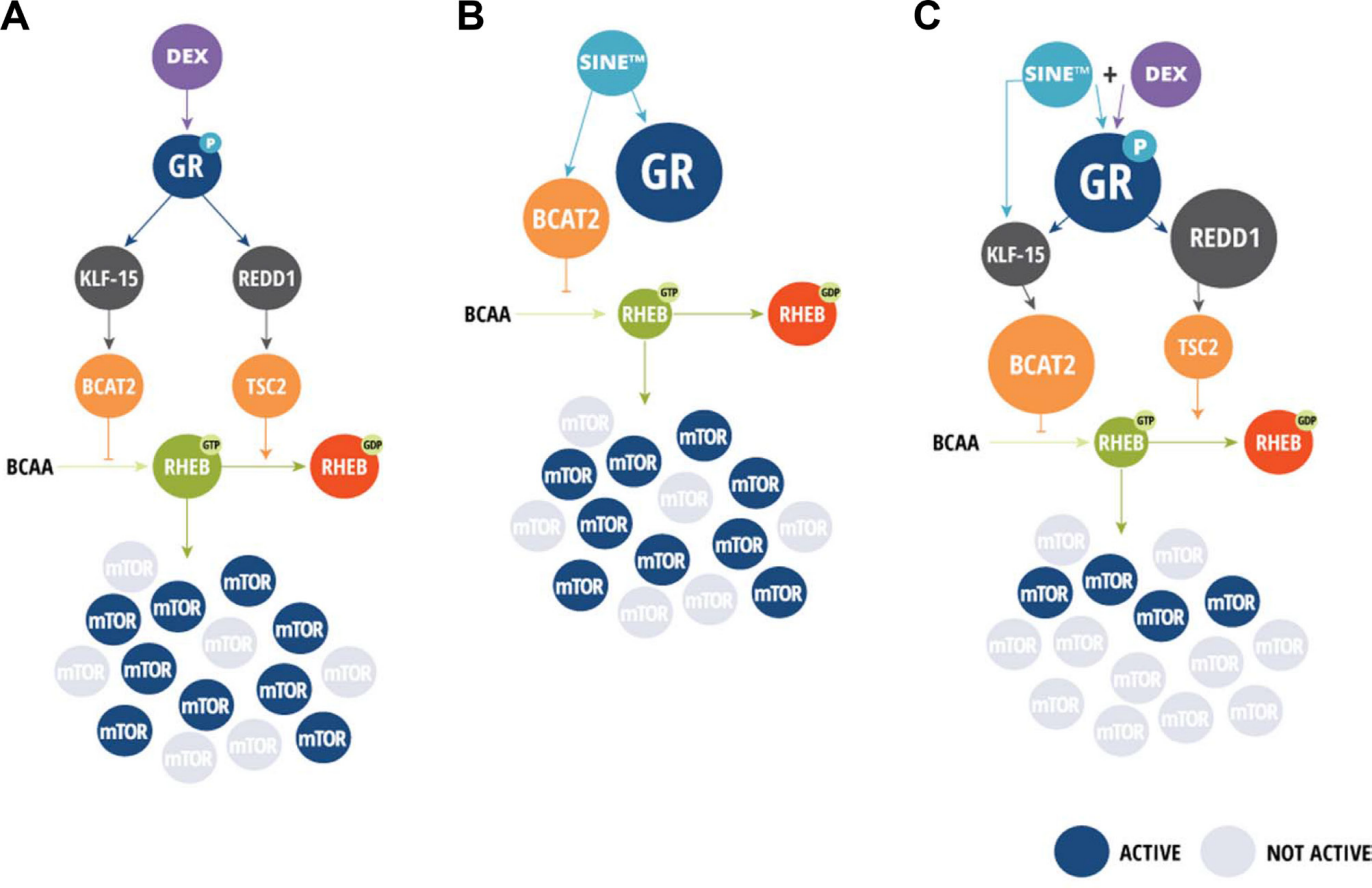
SEL-DEX proposed mechanism (**A**) DEX binds GR which is then phsopho-activated. Active GR induces the transcription of REDD1 and BCAT2 (indirectly) which marginally inhibits mTOR pathway activity. (**B**) Selinexor increases expression of GR and BCAT2. BCAT2 degrades branch chain amino acids (BCAA) resulting in mTOR inhibition. GR is not activated. (**C**) The SEL-DEX combination synergistically inhibits mTOR activity.

In conclusion (Figure 7), we found that the synergy of SEL-DEX can be explained by selinexor-induced increase in total GR levels, making cells more sensitive to DEX treatment, while remaining sensitive to cell death by XPO1 inhibition. Moreover, we show that SEL-DEX inhibits mTOR activity, a key contributor to myeloma progression. We further show that the inhibitory effect on mTOR activity in myeloma cells is partly through the expression of REDD1, a well-established regulator of mTOR activity, which may prove useful in predicting and monitoring responses in patients with MM receiving selinexor and DEX. Finally, the SEL-DEX combination is also effective in myeloma xenograft models and clearly shows a significant benefit over single agent treatment, providing compelling mechanistic rationale for future clinical studies.

## MATERIALS AND METHODS

### Cell culture

MM.1S (ATCC#CRL-2974), MM.1R (ATCC#CRL-2975), and H929 (ATCC#CRL-9068) MM cells were cultured in Roswell Park Memorial Institute 1640 media (Corning#15-040-CV) supplemented with heat inactivated 10% fetal bovine serum (Corning#35-011-CV), 100 units/mL penicillin, 100 μg/mL streptomycin (Gibco#15140122). Cells were maintained at 37°C with 5% CO_2_ in a humidified incubator.

### Viability assays

2 × 10^4^ MM.1S, MM.1R, and H929 cells/well in a 96-well plate were treated with various concentrations of selinexor, and DEX and incubated at 37°C in a 5% humidified CO_2_ incubator for 72 hours. Cell viability was determined using CellTiter-Fluor Viability Assay (Promega#G6082) and Caspase 3/7 Glo Viability Assay (Promega#G8092) per the manufacturer’s instructions. IC_50_ values were calculated using XLfit software.

### Nuclear/cytoplasmic subcellular fractionation assay

MM1.S and MM.1R cells were treated with selinexor, DEX, or both drugs for 4 hours. The cells were collected, washed with 1XPBS, and fractionation was carried out using the NE-PER nuclear and cytoplasmic extraction kit (Thermo Scientific#78833). Fractionation efficiency was evaluated by protein expression of subcellular marker proteins; GAPDH (cytoplasmic) and Lamin B (nuclear).

### Immunofluorescence

For the detection of phospho GR and total GR localization, MM.1S cells were treated according to the study design and washed with 1XPBS. Cells were plated to coverslips using cytospin. Cells were fixed with 100% ice-cold methanol (MeOH) and permeabilized/ blocked with 0.1% Tween 20, 0.3 M glycine, and 1% BSA in 1XPBS. The cells were then probed with the appropriate primary antibody for 1 hour and washed 3 times. The rabbit secondary antibody, Alexa Fluor 488 (Invitrogen, A11008) was used for all the staining, while nuclei were stained with DAPI (Invitrogen). Protein localization was visualized with a Nikon Eclipse Ti inverted fluorescence microscope (Nikon) and monochrome camera (ANDOR) at 20× magnification.

### GR transcriptional activity assay

MM.1S and MM.1R were treated with 1 μM selinexor and 100 nM DEX for 4 hours. Nuclear fractions were extracted from treated samples samples using a Nuclear Extract Kit (Cat# AY2002, Affymetrix) per the manufacturer’s instructions. The nuclear extract was examined for GR transcriptional activity using a GR DNA binding kit (Cat# EK1060, Affymetrix).

### qPCR

MM.1S and MM.1R cells were seeded in 6-well plates and treated with DEX and selinexor alone and in combination. Cells were collected and washed with 1XPBS. RNA was then extracted using the RNAeasy kit (QIAGEN). RNA was quantified and converted to cDNA. Gene expression levels were analyzed using the Viia7 with Taqman probes against the indicated genes. Gene expression was determined using GAPDH as an internal control.

### Western blot analysis

1.5 × 10^6^ cells were seeded in 6-well plates and treated with DEX and selinexor alone and in combination. The cells were then washed with 1X PBS and lysed with RIPA buffer (#89901, Thermo Scientific) supplemented with protease inhibitors (#05892791001, Roche) and phosphatase inhibitors (#04906837001, Roche). Protein levels were determined and normalized using Pierce’s BCA assay (#23225, Thermo Scientific). 20 μg of each sample were run in 4–12% Bis-Tris Gel (Life Technologies) and later transferred to nitrocellulose membrane using iBlot Gel Transfer Kit (Life Technologies). The membranes were blocked using LI-COR blocking buffer (#927-40000, LI-COR), probed with the indicated antibodies and analyzed using Licor Odyssey. Densitometry values were obtained using Image J software [72].

### TaqMan gene assay and antibodies

Real time PCR Taqman gene probes were purchased from Life Technologies (Carlsbad, CA, USA) (XPO1: Hs00418963_m1; GR: Hs00353740_m1; REDD1: Hs01111686_g1; KLF15: Hs00362736_m1; BCAT2: Hs01553550_m1; SESN2: Hs00230241_m1; DEPTOR: Hs00961900_m1; MNK2: Hs00179671_m1). The antibodies for Caspase-7 (#12827), PUMA (#4976), Bcl-2 (#2870), Bcl-XL (#2764), Glucocorticoid Receptor (#12041), Phospho (Ser-211) Glucocorticoid Receptor (#4161), Phospho (Thr-389) p70S6K (#9234), p70S6K (#2708), Phospho (Ser-235/S236) RPS6 (#4858), RPS6 (#2217), Phospho (Ser-65) 4EBP1 (#9456), 4EBP1 (#9644), BCAT2 (#9432), REDD1 (#2516), DEPTOR (#11816), SESN2 (#8487), RHEB (#13879) were purchased from Cell Signaling (Danvers, MA, USA). Antibodies targeting XPO1 (#sc-5595), Mcl-1 (#sc-819) and beta-Actin (#sc-81178) were purchased from Santa Cruz Biotechnology. The secondary antibodies for western blotting were purchased form LI-COR (Lincoln, NE, USA) and the secondary antibodies for immunofluorescence were purchased from Life Technologies (#A11008).

### Silencing assay

MM.1S and MM.1R cell lines were transfected with 40 nM of REDD1 (Thermo Scientific#s29166) and BCAT2 (Thermo Scientific#s1904) siRNA or 40 nM BLOCK-iT using Neon Transfection System (#MPK5000, Invitrogen). The transfection was performed using antibiotic free RPMI-1640 media using the condition (Voltage – 1500V, Width – 10ms, Pulses – 3). 48 hours post-transfection, the cells were treated with 200 nM selinexor and 100 nM DEX for 24 hours. The assay was performed in duplicate.

### Xenograft study

36 NOD SCID mice were inoculated subcutaneously in the left flank with 4 × 10^6^ H929 cells. Treatment was initiated when the tumors reached a mean volume of 109.5 mm^3^ (standard deviation ± 31 mm^3^ range 59–178 mm^3^). Mice were allocated to four 4 groups of nine mice such that mean tumor volume in each group was within the range of 108 to 113 mm^3^. Mice were treated with vehicle, DEX, selinexor, or a combination of DEX and selinexor. DEX was given daily via intra-peritoneal (IP) injection, while selinexor was given via oral gavage (PO) on Mondays, Wednesdays and Fridays. Animal weights and conditions were recorded daily, and tumors were measured on Mondays, Wednesdays and Fridays. The mice were euthanized on Day 29 and the tumors collected and preserved in formalin.

## SUPPLEMENTARY MATERIALS FIGURES AND TABLES


