# Supplementary material for: Selinexor synergizes with dexamethasone to repress mTORC1 signaling and induce multiple myeloma cell death
Source: Oncotarget. 2018 May 22;9(39):25529–44. doi: 10.18632/oncotarget.25368 (PMC5986633; doi:10.18632/oncotarget.25368)
Supplement: Supplementary file 1 [file oncotarget-09-25529-s001.pdf]

Selinexor synergizes with dexamethasone to repress mTORC1 signaling and induce multiple myeloma cell death

SUPPLEMENTARY MATERIALS

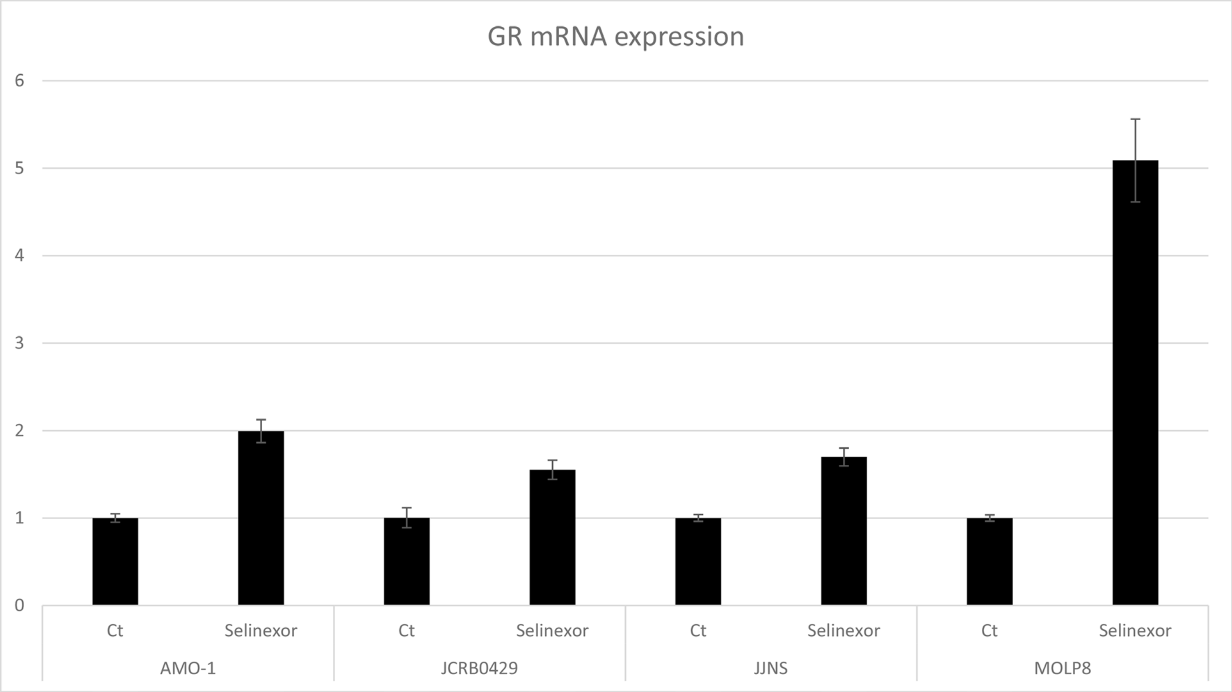

Supplementary Figure 1: Multiple myeloma cells were treated with 500 nM selinexor for 24 hours. The mRNA expression of the glucocorticoid receptor (NR3C1) was evaluated by real time PCR. Selinexor treatment induces the expression of GR across multiple myeloma cell lines.

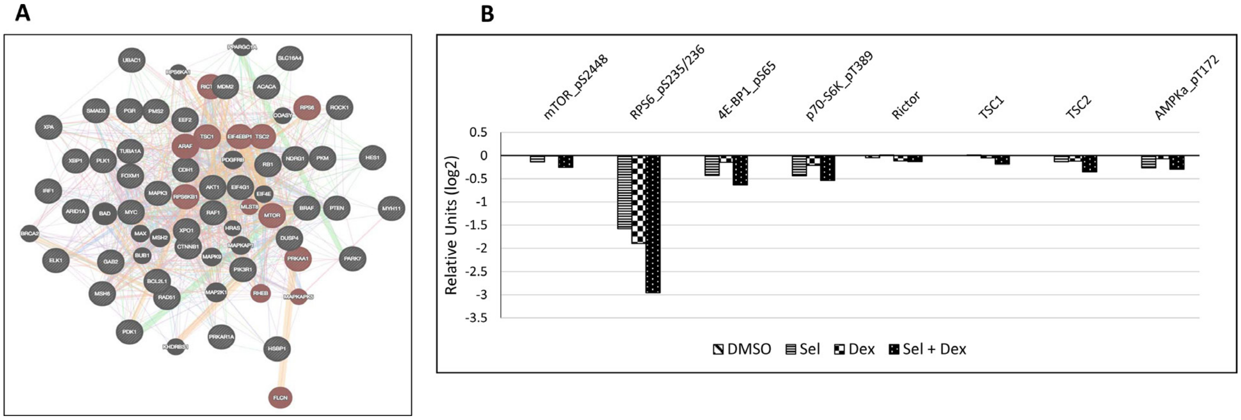

Supplementary Figure 2: (**A**) Cellular lysates of MM.1S cells treated with 200 nM selinexor and/or 100 nM DEX for 24 hours were tested by Reverse Phase Protein Array (RPPA) for the expression levels of 300 proteins. Ingenuity Pathway Analysis (IPA) of 64 proteins (grey circles) differentially expressed between the combination and single agents revealed reduction in the expression levels of mTOR related proteins (red circles). (**B**) Graphical representation of the changes in the expression levels of the mTOR related proteins (red circles in Figure 3A) by selinexor and DEX alone or in combination compared to the DMSO treated sample.

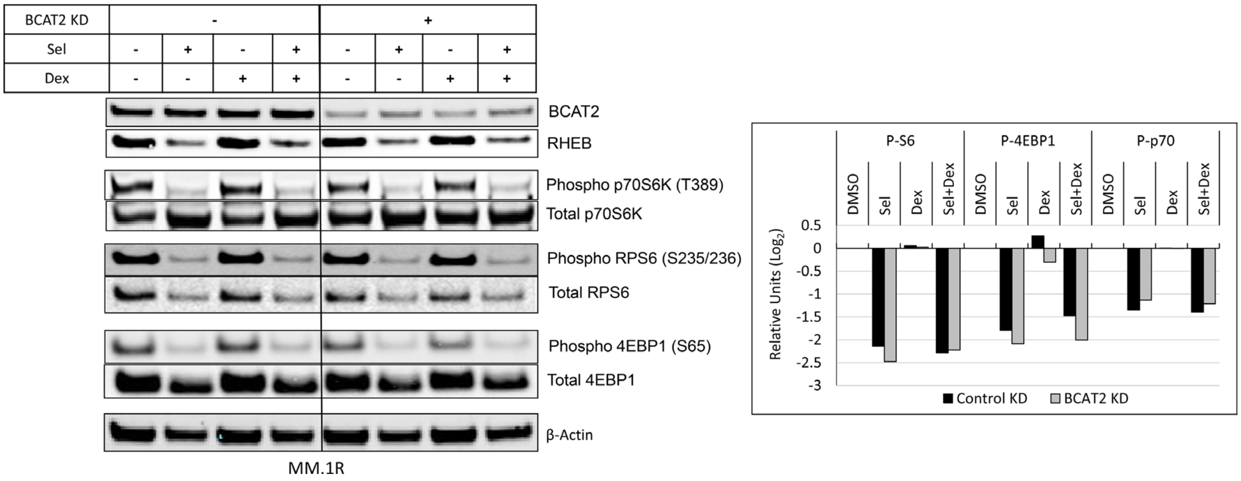

Supplementary Figure 3: MM.1R cells were transfected with either 40 nM BCAT2 or control siRNA using Neon transfection system and the cells were treated for 24 hours with 200 nM selinexor and 100 nM DEX for 48 hours post transfection. Densitometry analysis showed BCAT2 silencing didn't significantly affect the impact of selinexor and DEX on mTOR targets.

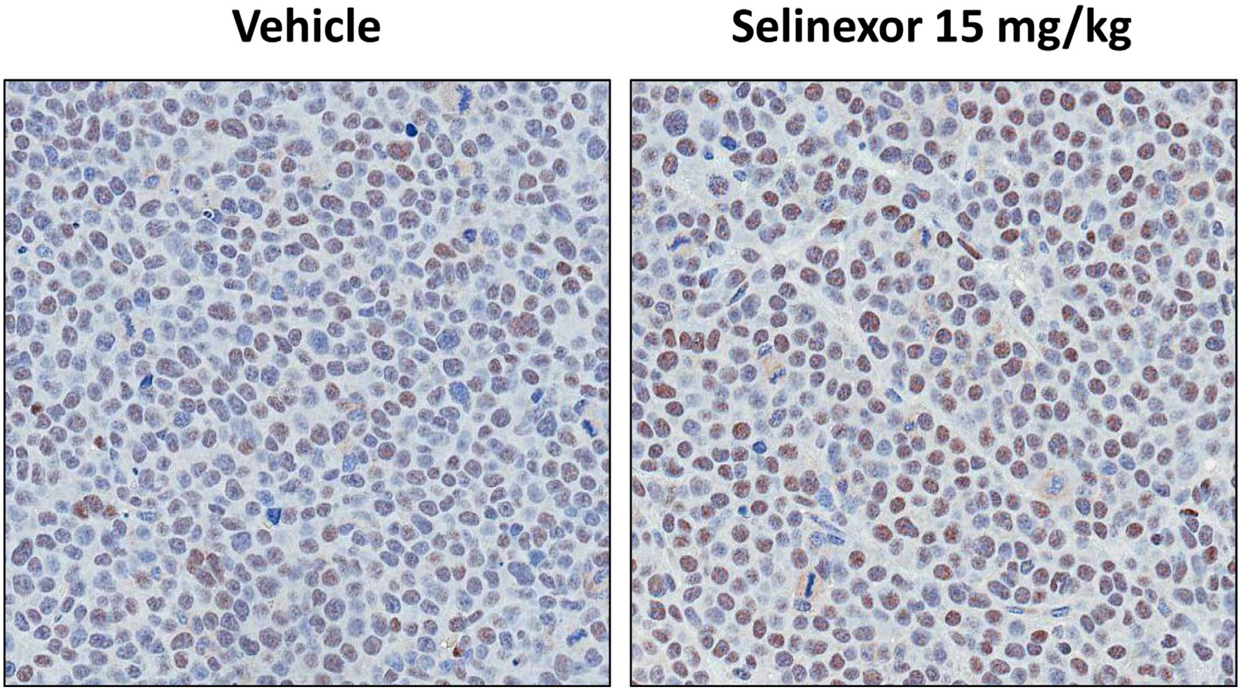

Supplementary Figure 4: NOD-SCID mice were inoculated with MM.1S cells until the mean tumor volume in each group was within the range of 108 to 113 mm^3. Mice were treated with vehicle or 15 mg/kg of selinexor for 18 days. Selinexor was given via oral gavage on a Monday-Wednesday-Friday schedule (MWF). The group that received vehicle exhibited basal levels of GR expression when compared to the selienxor treated group, which exhibited an induction of GR expression levels.

Supplementary Table 1: MM.1S CI data for non-constant combination

Dose sel	Dose dex	Effect	CI
333.3	0.39	0.8638	0.56703
333.3	1.56	0.8873	0.44375
333.3	6.25	0.9029	0.37628
333.3	25.0	0.9229	0.31442
333.3	100.0	0.9402	0.33398
111.1	0.39	0.7275	0.52064
111.1	1.56	0.7458	0.47456
111.1	6.25	0.81	0.33018
111.1	25.0	0.8864	0.21587
111.1	100.0	0.9367	0.21635
37.04	0.39	0.4758	0.61633
37.04	1.56	0.5418	0.47262
37.04	6.25	0.6642	0.30870
37.04	25.0	0.7987	0.23897
37.04	100.0	0.8985	0.28272
12.35	0.39	0.2718	0.59169
12.35	1.56	0.356	0.41607
12.35	6.25	0.5053	0.29735
12.35	25.0	0.6772	0.31731
12.35	100.0	0.8413	0.41979
4.12	0.39	0.1294	0.60712
4.12	1.56	0.1998	0.42242
4.12	6.25	0.3855	0.29741
4.12	25.0	0.5791	0.40390
4.12	100.0	0.7666	0.64054
1.37	0.39	0.0505	0.74712
1.37	1.56	0.1225	0.40582
1.37	6.25	0.2744	0.37768
1.37	25.0	0.5114	0.49082
1.37	100.0	0.7206	0.79659
0.46	0.39	0.01	2.08851
0.46	1.56	0.0314	1.21234
0.46	6.25	0.2197	0.44870
0.46	25.0	0.4923	0.51490
0.46	100.0	0.6902	0.91253
0.15	0.39	0.01	1.11921
0.15	1.56	0.0748	0.39042
0.15	6.25	0.2293	0.40642
0.15	25.0	0.4784	0.53838
0.15	100.0	0.685	0.93230

2×10^4 MM.1S cells/well in a 96-well plate were treated with various concentrations of selinexor, and DEX and incubated at 37°C in a 5% humidified CO_2 incubator for 72 hours. Cell viability was determined using CellTiter-Fluor Viability Assay (Promega#G6082) and Caspase 3/7 Glo Viability Assay (Promega#G8092) per the manufacturer's instructions. CI values were calculated using CI software. The values obtained show that selinexor and dexamethasome synergize to induce cell death at most of the concentrations tested.

Supplementary Table 2: H929 CI data for non-constant combination

Dose sel	Dose dex	Effect	CI
10000.0	10000.0	0.881	1.40273
10000.0	3333.3	0.834	2.18234
10000.0	1111.1	0.848	1.93655
10000.0	370.4	0.879	1.43320
10000.0	123.5	0.816	2.51574
3333.3	10000.0	0.834	0.72789
3333.3	3333.3	0.818	0.82612
3333.3	1111.1	0.833	0.73344
3333.3	370.4	0.811	0.87071
3333.3	123.5	0.818	0.82589
370.4	10000.0	0.794	0.11053
370.4	3333.3	0.722	0.17289
370.4	1111.1	0.733	0.16138
370.4	370.4	0.732	0.16206
370.4	123.5	0.724	0.16961
123.5	10000.0	0.683	0.07832
123.5	3333.3	0.557	0.14477
123.5	1111.1	0.614	0.10221
123.5	370.4	0.551	0.13610
123.5	123.5	0.578	0.11899
41.2	10000.0	0.573	0.07467
41.2	3333.3	0.427	0.15335
41.2	1111.1	0.464	0.08209
41.2	370.4	0.435	0.08373
41.2	123.5	0.436	0.07839
13.7	10000.0	0.472	0.14602
13.7	3333.3	0.365	0.20495
13.7	1111.1	0.368	0.08912
13.7	370.4	0.34	0.06682
13.7	123.5	0.326	0.05399
4.6	10000.0	0.442	0.19142
4.6	3333.3	0.325	0.31262
4.6	1111.1	0.301	0.15831
4.6	370.4	0.309	0.05774
4.6	123.5	0.34	0.02237
1.5	10000.0	0.389	0.37076
1.5	3333.3	0.214	1.84074
1.5	1111.1	0.268	0.24419
1.5	370.4	0.252	0.11041
1.5	123.5	0.316	0.01757

2×10^4 H929 cells/well in a 96-well plate were treated with various concentrations of selinexor, and DEX and incubated at 37°C in a 5% humidified CO_2 incubator for 72 hours. Cell viability was determined using CellTiter-Fluor Viability Assay (Promega#G6082) and Caspase 3/7 Glo Viability Assay (Promega#G8092) per the manufacturer's instructions. CI values were calculated using Compusyn software. Although H929 cells are somewhat resistant to dexamethasome as a single agent, the values obtained show that selinexor and dexamethasome synergize to induce cell death.
